# Preclinical evaluation of ^225^Ac-labeled minigastrin analog DOTA-CCK-66 for Targeted Alpha Therapy

**DOI:** 10.1007/s00259-024-06927-z

**Published:** 2024-10-11

**Authors:** Nadine Holzleitner, Meryl Vilangattil, Abir Swaidan, Clara Diaz Garcia-Prada, Marco F. Taddio, Pauline Jeanjean, Christine E. Mona, Constantin Lapa, Angela Casini, Thomas Günther, Giuseppe Carlucci

**Affiliations:** 1 Chair of Pharmaceutical Radiochemistry, Department of Chemistry, School of Natural Sciences, Technical University of Munich, Walther-Meissner-Str. 3, 85748 Garching, Germany; 2 Department of Molecular and Medical Pharmacology, Biomedical Cyclotron Facility, University of California Los Angeles, 780 Westwood Plaza, Los Angeles, CA 90024 USA; 3 Nuclear Medicine, Faculty of Medicine, University of Augsburg, Augsburg, Germany; 4 Chair of Medicinal and Bioinorganic Chemistry, Department of Chemistry, School of Natural Sciences, Technical University of Munich, Garching, Germany; 5 Molecular Imaging Program at Stanford (MIPS), Department of Radiology, School of Medicine, Stanford University, Stanford, CA USA

**Keywords:** CCK-2R, MTC, Actinium-225, Targeted Alpha Therapy (TAT)

## Abstract

**Abstract:**

The recently developed metabolically more stable minigastrin derivative, DOTA-CCK-66, displayed promising preclinical data when labeled either with ^68^Ga or ^177^Lu. First positron emission tomography/computed tomography (PET/CT) imaging using [^68^Ga]Ga-DOTA-CCK-66 in two patients suffering from medullary thyroid carcinoma (MTC) displayed a favorable biodistribution profile. Here, we aim to investigate the therapeutic potential of [^225^Ac]Ac-DOTA-CCK-66 as a targeted α-therapy (TAT) agent in a comparative treatment study of [^177^Lu]Lu- versus [^225^Ac]Ac-DOTA-CCK-66.

**Methods:**

Treatment studies were performed (3 groups, n = 5, AR42J tumor-bearing 394-NOD SCID mice). Control group animals were injected with [^68^Ga]Ga-DOTA-CCK-66 (1.1 MBq, PET/CT imaging), while treatment group animals received a single dose of either [^177^Lu]Lu-DOTA-CCK-66 (37 MBq, radioligand therapy (RLT)) or [^225^Ac]Ac-DOTA-CCK-66 (37 kBq, TAT). All animals' tumor volume and body weight were monitored twice a week until end-point criteria were reached. Blood samples were evaluated (VetScan VS2, Abaxis) once mice were sacrificed.

**Results:**

Upon treatment, an initial decline in tumor volume, followed by a significantly delayed tumor growth of treated cohorts, was observed. Mean survival of ^177^Lu- as well as ^225^Ac-treated animals was increased by 3- (37 ± 3 d) and 4.5-fold (54 ± 6 d), respectively, when compared to non-treated animals (12 ± 3 d). Blood sample analysis did not indicate toxic side effects to the liver, kidney, or stomach upon ^177^Lu and ^225^Ac-treatment.

**Conclusion:**

We demonstrated a substantial therapeutic efficacy of ^177^Lu- and ^225^Ac-labeled DOTA-CCK-66. As expected, treatment with the latter resulted in the highest mean survival rates. These results indicate a high therapeutic potential of ^225^Ac-labeled DOTA-CCK-66 for TAT in MTC patient management.

**Graphical abstract:**

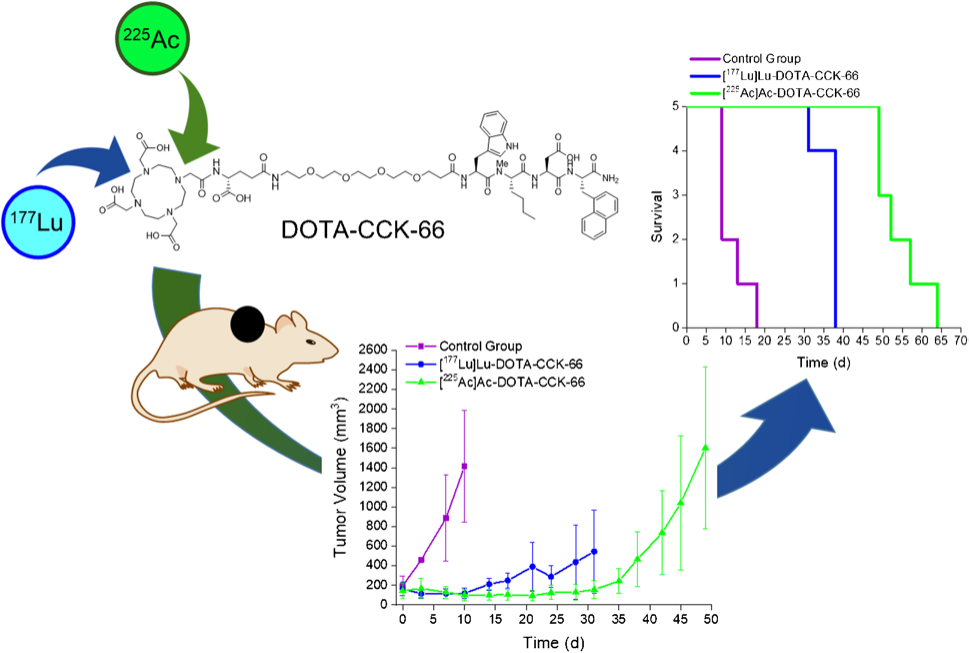

**Supplementary Information:**

The online version contains supplementary material available at 10.1007/s00259-024-06927-z.

## Introduction

Recently, the FDA approved radiopharmaceuticals Lutathera® (targets the somatostatin type 2 receptor (sst2r), 2018) and Pluvicto® (targets the prostate-specific membrane antigen (PSMA), 2022), two ^177^Lu-labeled compounds addressing different molecular targets and indications. The field of peptide-based compounds for radioligand therapy (RLT) has provided significant breakthroughs and has since been rapidly progressing as a viable option for cancer treatment [1, 2]. Besides β^¯^-emitting radionuclides routinely applied for RLT such as ^177^Lu and ^131^I, α-particle emitting radioisotopes are emerging with first clinical trials completed or ongoing delivering impressive results (e.g., [^225^Ac]Ac-PSMA-617 and [^212^Pb]Pb-DOTAMTATE) [3–5]. Especially the higher linear energy transfer obtainable when using α- instead of β^¯^-emitting radionuclides (60–230 keV/µm versus 0–2 keV/µm) has been demonstrated to increase therapeutic efficacies [6–10]. One prominent example of α-particle emitting radionuclides is ^225^Ac, which comprises a half-life of 9.9 d and undergoes four rapid consecutive α-particle emissions in its decay chain to a stable ^209^Bi [11]. Despite the promise of α-emitters, poor availability, complex dosimetry calculations, and the high recoil energy present during the emission of an α-particle, which often leads to the dissociation of the radiometal from the chelating unit, represent challenges of such radionuclides that still need to be overcome [11–13].

Apart from the radionuclide, it is important to define a suitable target and compound for RLT. The cholecystokinin-2 receptor (CCK-2R), which is overexpressed in a variety of cancer types, such as MTC (92%), small cell lung cancer (57%), astrocytoma (65%) and stromal ovarian cancer (100%), was reported to be a promising target for RLT [14–17]. Since then, research mainly focused on addressing the CCK-2R for imaging and therapy of MTC, a cancer type for which, only limited therapeutic options (e.g., tyrosine kinase inhibitors) are available apart from surgery. To date, a 10-year survival rate of approximately 40% for patients suffering from metastatic or recurrent disease is achieved [18–20]. Thus, RLT could significantly improve patient management. Currently, three CCK-2R targeting peptides ([^111^In]In-CP04, [^177^Lu]Lu-DOTA-PP-F11N and [^68^Ga]Ga-DOTA-MGS5) are being evaluated in phase I/II clinical trials, while only [^177^Lu]Lu-DOTA-PP-F11N is tested in a therapeutic context [21–23].

Recently, our group reported preclinical evaluations on the theranostic (^68^Ga/^177^Lu pair) minigastrin analog, DOTA-CCK-66 (DOTA-γ-glu-PEG_3_-Trp-(N-Me)Nle-Asp-1-Nal-NH_2_, PEG: polyethylene glycol), which displayed enhanced in vivo stability compared to previously published minigastrin derivatives and thus, favorable biodistribution profiles at 1 h and 24 h after injection [24]. First proof-of-concept [^68^Ga]Ga-DOTA-CCK-66 PET/CT imaging in patients suffering from metastatic MTC revealed high tracer accumulation in lesions and no significant uptake in healthy organs except in the stomach (endogenously expressing CCK-2R) and the kidneys (tracer excretion). These promising data suggest further evaluation of DOTA-CCK-66 in a therapeutic setting [24, 25].

Hence, within this study, we aimed to assess wether ^225^Ac-directed TAT is a suitable or even more effective alternative to ^177^Lu-RLT for CCK-2R targeted applications using DOTA-CCK-66 as a precursor. Therefore, we completed a comparative single-dose preliminary proof-of-concept treatment approach in AR42J tumor-bearing mice of [^225^Ac]Ac-DOTA-CCK-66 and [^177^Lu]Lu-DOTA-CCK-66. Potential adverse effects of [^225^Ac]Ac-DOTA-CCK-66 or [^177^Lu]Lu-DOTA-CCK-66, respectively, on the kidneys, the stomach, and the liver were also investigated.

## Materials and methods

### Chemical synthesis and labeling procedures

Synthesis of DOTA-CCK-66 was carried out as previously published [24]. A brief characterization of the peptide is provided in the Supplementary Information (Supplemental Fig. 1). ^68^Ga-labeling of DOTA-CCK-66 was completed at 90 °C for 5 min. ^225^Ac was supplied by Oak Ridge National Laboratory (Oak Ridge, TN, USA). ^225^Ac-labeling was carried out at 90 °C for 30 min. Synthesis of the ^177^Lu-labeled peptide was performed at 95 °C for 5 min. Radiochemical purities (RCPs) of all radiolabeling procedures were determined via radio-thin layer chromatography (TLC). A detailed description of all radiolabelings can be found in the Supplementary Information.

### In vitro experiments

In vitro stability studies were conducted 1–10 d after incubation of [^225^Ac]Ac-DOTA-CCK-66 at 37 °C in human serum (*n* = 3), in analogy to a previously published protocol [24]. Lipophilicity depicted as log*D*_7.4_ value of the ^225^Ac-labeled compound was determined using an established protocol [24]. For our preliminary treatment study, we chose to use AR42J cells for better comparison of treatment data with our previous biodistribution data collected with the same tumor model [24]. CCK-2R expression of AR42J cells was verified by flow cytometry using a polyclonal rabbit CCK antibody (1 mg/mL, ThermoFisher Scientific Inc., Waltham, MA, USA). Samples were measured on a LSRII flow cytometer (BD) and analyzed using FlowJo (Tree Star Inc., Ashland, OR, USA). Detailed information on all in vitro experiments can be found in the Supplementary Information.

### In vivo experiments

Female 394-NOD SCID mice (6–8 weeks old, Charles River Laboratories, Wilmington, MA, USA) were housed under pathogen-free conditions and treated according to the Universtiy of California, Los Angeles Animal Research Committee protocol guidelines. The study was performed in compliance with the ARRIVE guidelines (Animal Research: Reporting of In Vivo Experiments). No masking was applied in the allocation of the experiments.

AR42J cells (2 × 10^6^ cells, 100 µL suspended in Matrigel) were subcutaneously inoculated into the right shoulder region of mice. When tumors reached 30 to 290 mm^3^ (14 to 17 d after inoculation), animal cages performed randomisation into 3 groups (5 per group). On day 0 of the experiment mice received a single-dose injection of either [^68^Ga]Ga-DOTA-CCK-66 (1.1 MBq, PET/CT imaging, control cohort), [^177^Lu]Lu-DOTA-CCK-66 (37 MBq) or [^225^Ac]Ac-DOTA-CCK-66 (37 kBq). Tumor volume (caliper measurements, length^2^ × width/2) and body weight of all animals were subsequently monitored twice a week. Animals were sacrificed after reaching one of the end-point criteria (weight loss of more than 20%, tumor volume of more than 2,000 mm^3^, ulceration of the tumor, respiratory distress, or change of behavior).

Static PET/CT images of control group animals were acquired (*n* = 5) 1 h after injection of [^68^Ga]Ga-DOTA-CCK-66 using a preclinical PET/CT scanner (Genisys 8 PET/CT; Sofie Biosciences, Dulles, VA, USA), according to a published procedure [26]. PET image reconstruction was carried out using a maximum-likelihood expectation maximization with 60 iterations. Correction for photon attenuation of all images was performed. Acquisition parameters for CT applications were 40 kVp and 190 mA. Acquired data were analyzed using OsiriX Imaging Software (version 3.9.3; Pixmeo SARL, Bernex, Switzerland) [26].

Hematological analysis (aspartate aminotransferase (AST); alanine aminotransferase (ALT); Na^+^; alkaline phosphatase (ALP); total protein (TP); albumin (ALB); bicarbonate (^*t*^CO_2_); total bilirubin (TBIL); hemoglobulin (GLOB); glucose (GLU); blood urea nitrogen (BUN); K^+^; Ca^2+^; VetScan VS2, Abaxis, Union City, CA, USA) of treated and non-treated AR42J tumor-bearing animals was performed. Hence, blood samples were collected retro-orbitally once an end-point criterium was reached. In addition, blood samples of [^177^Lu]Lu- or [^225^Ac]Ac-DOTA-CCK-66 treated naïve 394-NOD SCID mice were collected and analyzed in analogy to their treated tumor-bearing counterparts. A detailed description of the study protocol can be found in the Supplementary Information.

For immunohistochemistry (IHC) as well as hematoxylin and eosin (HE) staining, different organs from the [^225^Ac]Ac-DOTA-CCK-66 treated (kidneys, liver, muscle, stomach and tumor) as well as control group animals (kidneys, liver, muscle and stomach) were collected once the animals reached one of the end-point criteria, and fixed in 10% formalin for 48 h. Organs of three mice per cohort (*n* = 5) were isolated. Mice were randomly chosen for organ collection. Thereafter, tissue samples were transferred to 70% ethanol and stored until radioactivity decayed (approximately 4–6 months). Samples were analyzed using a Leica Bond RX processor (Leica Biosystems, Deer Park, TX, USA). A polyclonal rabbit CCK antibody was used as the primary antibody (1 mg/mL, ThermoFisher Scientific Inc.). A detailed description of the protocol can be found in the Supplementary Information.

Acquired data were statistically analyzed using the Student’s *t-*test via Excel (Microsoft Corporation, Redmond, WA, USA) and OriginPro software (version 9.7) from OriginLab Corporation (Northampton, MA, USA). Acquired *P* values of less than 0.05 were considered statistically significant.

## Results

### Synthesis and radiolabeling

^225^Ac-labeling of the peptide precursor was performed manually, resulting in quantitative radiochemical yields (RCYs, > 99%, non-decay corrected) and high radiochemical purities (RCPs, (> 95%) with molar activities (A_m_) of 74 to 222 MBq/µmol. No further purification steps before usage were performed. ^68^Ga-labeling resulted in an RCP > 95% and A_m_ of 37 GBq/µmol. ^177^Lu-labeling was accomplished in an RCY > 99% (non-decay corrected), RCP > 95%, and A_m_ of 21 GBq/µmol. No optimization of radiolabeling strategies was performed. Reproducibility of ^225^Ac-labeling was verified (*n* ≥ 5).

### In vitro characterization

**Fig. 1:**
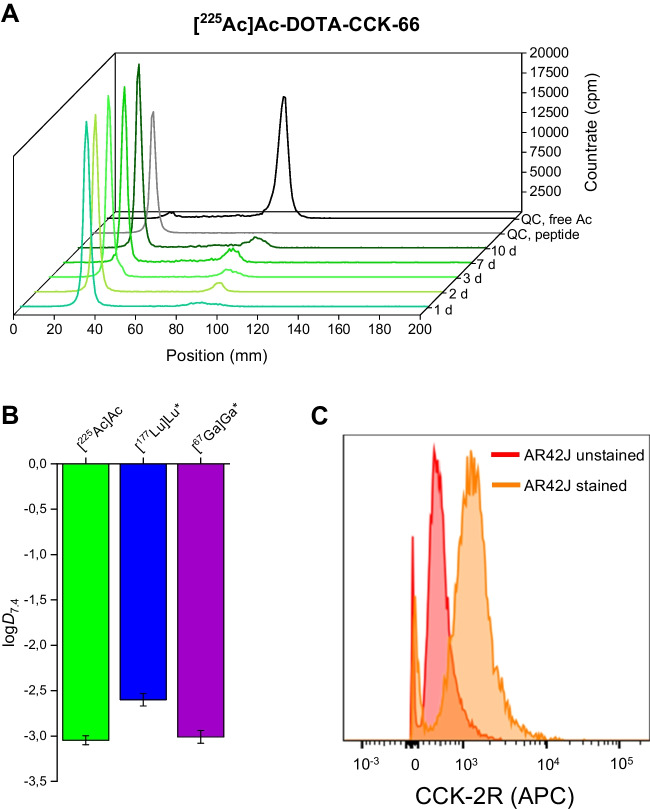
In vitro characterization of [^225^Ac]Ac-DOTA-CCK-66. **A** Stability in human serum after incubation at 37 °C for 1–10 days (*n* = *3*, depicted in green colors), as well as a quality control chromatogram of the ^225^Ac-labeled peptide (light grey) and a reference chromatogram of free ^225^Ac (dark grey). **B** Lipophilicity depicted as distribution coefficients at *pH* = 7.4 (log*D*_7.4_) of [^225^Ac]Ac-DOTA-CCK-66, [^177^Lu]Lu-DOTA-CCK-66 and [^67^Ga]Ga-DOTA-CCK-66. *Data taken from Günther et al. [24]. **C** CCK-2R expression of AR42J cells as determined by flow cytometry using a polyclonal CCK antibody

Evaluation of in vitro stability of [^225^Ac]Ac-DOTA-CCK-66 in human serum over time displayed > 90% intact peptide after up to 3 d of incubation at 37 °C (Fig. 1A, Supplemental Table 1). After 10 days, 83.4 ± 10.3% intact [^225^Ac]Ac-DOTA-CCK-66 were still observed. Lipophilicity of [^225^Ac]Ac-DOTA-CCK-66 was determined to a log*D*_7.4_ value of − 3.04 ± 0.05 (Fig. 1B). CCK-2R expression of AR42J cells was verified by flow cytometry (Fig. 1C).

### PET/CT imaging

**Fig. 2:**
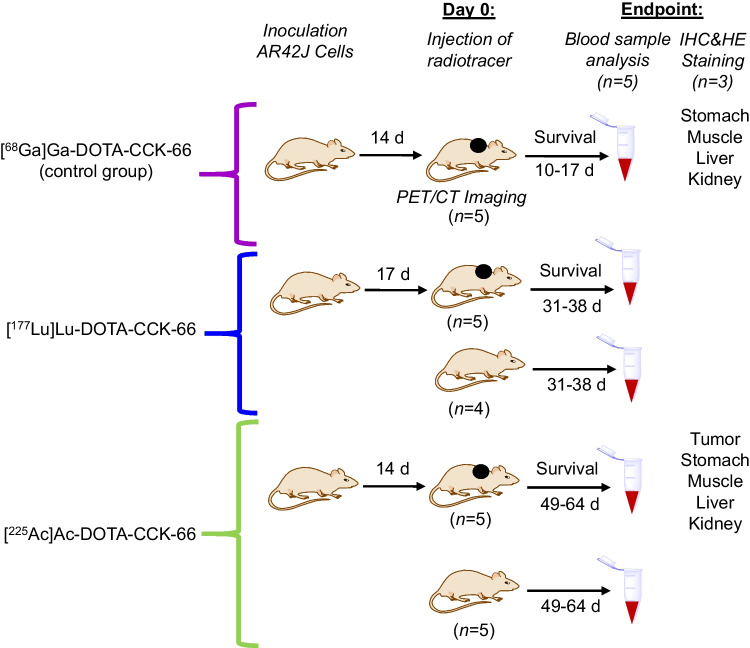
Study design of animal experiments (5 × 5 animals). AR42J tumors are depicted as black dots. Blood sample analysis of ^177^Lu- and ^225^Ac-treated tumor-bearing and naïve mice was performed

**Fig. 3:**
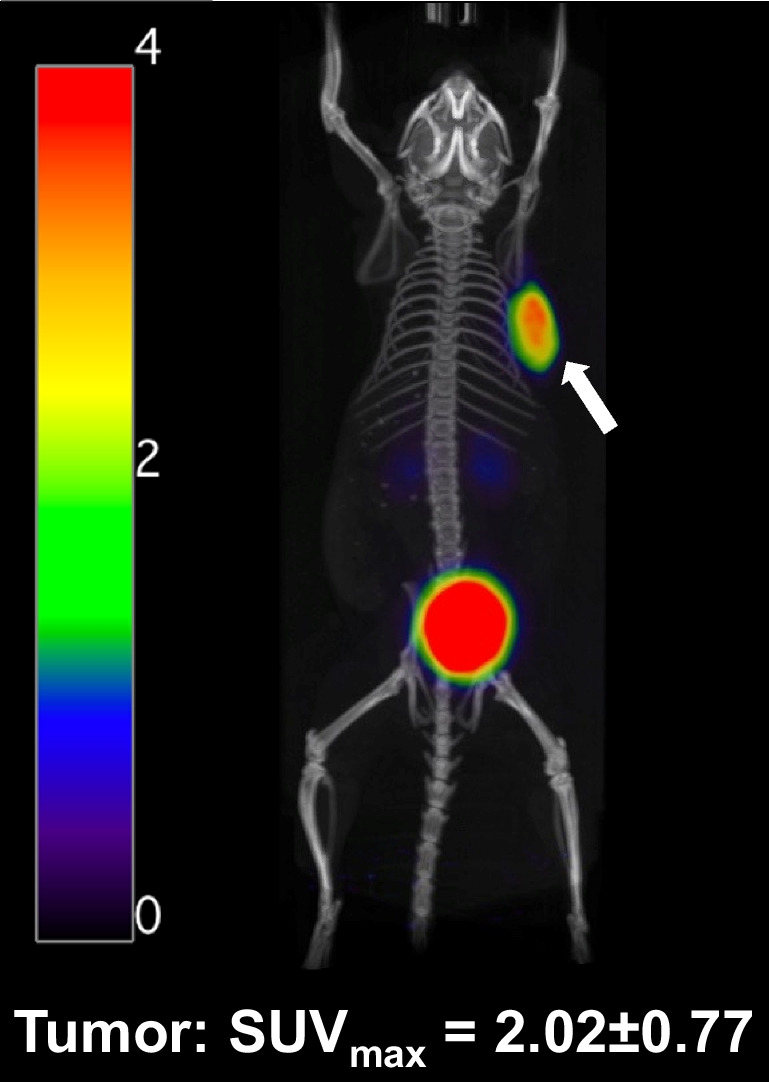
Representative PET/CT scan ([^68^Ga]Ga-DOTA-CCK-66; 1.1 MBq, 30 pmol) of a control group AR42J tumor-bearing mouse on day 0 of the experiment. Tumor is indicated with a white arrow

In total, 25 animals were used for PET/CT imaging (5, control cohort), treatment studies (3 × 5, AR42J tumor-bearing animals), hematological evaluations (5 × 5, all animals used) and IHC/HE staining (2 × 3, animals randomly selected from ^225^Ac-treated and control cohort) (Fig. 2). PET/CT imaging studies of AR42J tumor-bearing 394-NOD SCID mice at 1 h after injection of [^68^Ga]Ga-DOTA-CCK-66 (1.1 MBq, *n* = *5*) confirmed an increased tumor uptake in all five mice (SUV_max_ = 2.02 ± 0.77%), while overall non-target tissue uptake was low, with only one exception being the kidneys (Fig. 3, Supplemental Fig. 2).

### ^225^Ac-TAT versus ^177^Lu-RLT

**Fig. 4:**
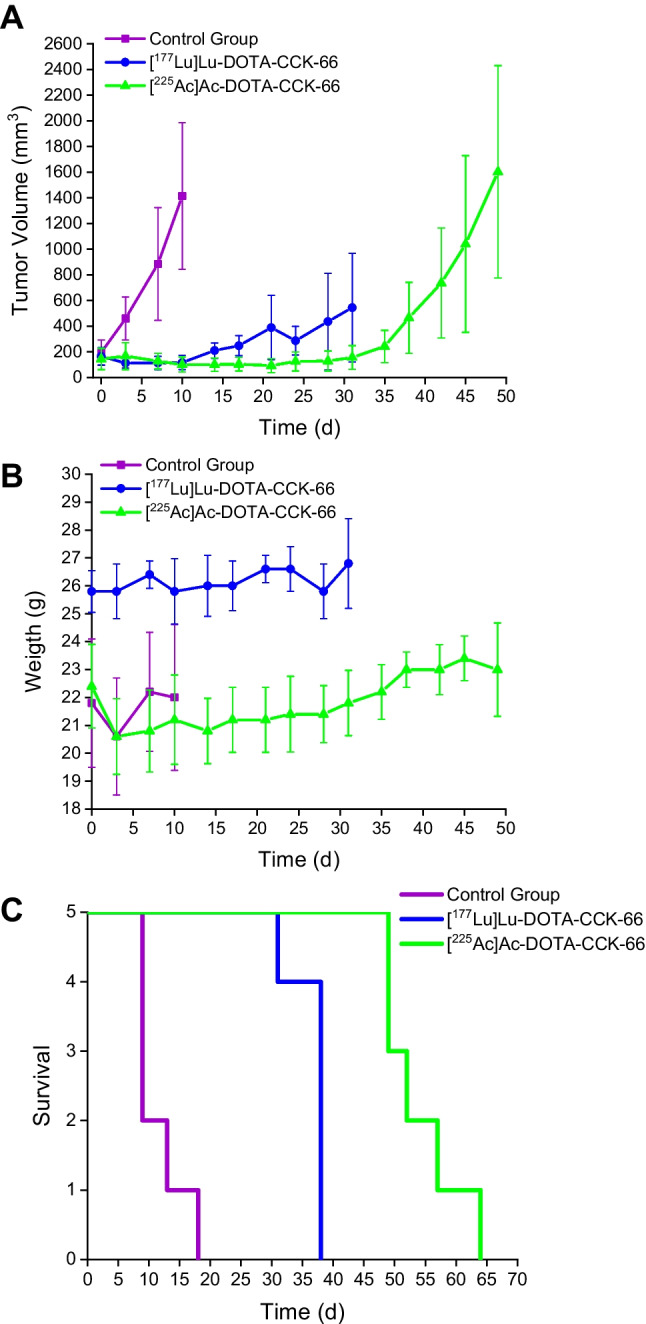
**A** Tumor growth inhibition, **B** body weight and (**C**) Kaplan-Meyer curves of AR42J tumor-bearing mice treated with [^225^Ac]Ac-DOTA-CCK-66 (37 kBq, *n* = 5) or [^177^Lu]Lu-DOTA-CCK-66 (37 MBq, *n* = 5), compared to untreated control (*n* = 5)

Equal tumor volumes (145 ± 40 mm^3^) were chosen for both treatment groups. Single dose administration of [^225^Ac]Ac-DOTA-CCK-66 (37 kBq), [^177^Lu]Lu-DOTA-CCK-66 (37 MBq) or [^68^Ga]Ga-DOTA-CCK-66 (1.1 MBq, control group) revealed an initial decline, followed by a decelerated tumor growth for the treated mice compared to the control cohort (Fig. 4, Supplemental Table 2). Thus, a 3- and 4.5-fold increase in mean survival of ^177^Lu- (37 ± 3 d) as well as ^225^Ac-treated mice (54 ± 6 d), respectively, as opposed to the control group animals (12 ± 3 d), was determined. In addition, a 1.5-fold increase in mean survival was observed for ^225^Ac-TAT compared to ^177^Lu-RLT (mean survival: 54 d versus 37 d, *p* = 0.0003). None of the animals from the experiment exhibited a weight loss of more than 20% of their initial body weight (day 0, Supplemental Table 3).

### Hematological analysis

**Fig. 5:**
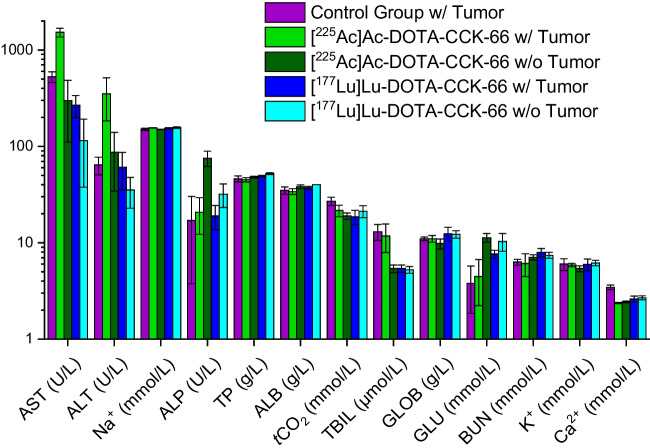
Blood values of untreated control (AR42J tumor, *n* = 5) versus AR42J tumor-bearing 394-NOD SCID treatment group mice ([^225^Ac]Ac-DOTA-CCK-66: 37 kBq, *n* = 5 and [^177^Lu]Lu-DOTA-CCK-66: 37 MBq, *n* = 5) as well as naïve animals injected either with [^225^Ac]Ac-DOTA-CCK-66 (37 kBq, *n* = 5) or [^177^Lu]Lu-DOTA-CCK-66 (37 MBq, *n* = 4)

Analysis of blood samples of control group (sacrificed on days 10 (*n* = 3), 14 and 17), treatment group and naïve mice injected with [^177^Lu]Lu-DOTA-CCK-66 (sacrificed on days 31 and 38 (*n* = 3)) and [^225^Ac]Ac-DOTA-CCK-66 (sacrificed on days 49 (*n* = 2), 52, 57 and 64) revealed that mean Na^+^ (150–157 mmol/L), TP (45–52 µmol/L), ALB (34–40 g/L), ^*t*^CO_2_ (19–22 mmol/L), GLOB (9.8–12.4 g/L), BUN (5.8–7.9 mmol/L), K^+^ (5.4–6.2 mmol/L) and Ca^2+^ (2.4–3.3 mmol/L) blood levels were in a similar range for all five animal cohorts evaluated (Fig. 5; Supplemental Tables 4 and 5). In contrast, AST values were noticeably differing between cohorts, with [^225^Ac]Ac-DOTA-CCK-66 treated (1713 ± 264 U/L versus 298 ± 187 U/L, *p* = 0.00001) or [^177^Lu]Lu-DOTA-CCK-66 treated (269 ± 69 U/L versus 115 ± 77 U/L, *p* = 0.013) AR42J tumor-bearing animals depicting higher values then naïve animals treated accordingly. In addition, AST values of tumor-bearing animals treated with [^225^Ac]Ac-DOTA-CCK-66, were significantly elevated compared to the untreated control (516 ± 68 U/L, *p* = 0.029) and [^177^Lu]Lu-DOTA-CCK-66 (*p* < 0.00001) treated mice. While ALT values of ^177^Lu-treated tumor-bearing (60.8 ± 25.4) and naïve mice (35.3 ± 12.4 U/L, *p* = 0.07) were similar, that of ^225^Ac-treated tumor-bearing (345 ± 166 U/L) and naïve animals (87.2 ± 52.9 U/L, *p* = 0.008) differed significantly. Furthermore, ALT values of AR42J tumor-bearing untreated control animals (79.2 ± 13.3 U/L) were comparable and noticeably decreased compared to [^177^Lu]Lu-DOTA-CCK-66 treated (*p* = 0.118) and [^225^Ac]Ac-DOTA-CCK-66 treated tumor-bearing animals (*p* = 0.0058), respectively. ^225^Ac-treated (20.8 ± 8.6 U/L, *p* = 0.148) and ^177^Lu-treated tumor-bearing animals (19.0 ± 5.3 U/L, *p* = 0.113) depicted comparable ALP values to the untreated control cohort (29.6 ± 13.2 U/L). However, ALP values of ^177^Lu-treated or ^225^Ac-treated tumor-bearing animals were significantly lower than those of their ^177^Lu-treated (32 ± 9 U/L, *p* = 0.034) or ^225^Ac-treated (76 ± 14 U/L, *p* = 0.00006) naïve counterparts. TBIL as well as GLU values of tumor-bearing untreated control (TBIL: 8.8 ± 2.5 µmol/L; GLU: 5.9 ± 1.9 mmol/L) and ^225^Ac-treated animals (TBIL: 11.8 ± 3.9 µmol/L; GLU: 4.5 ± 2.2 mmol/L) were found to be noticeably higher as well as lower, respectively, when compared to those of ^177^Lu-treated tumor-bearing (TBIL: 5.4 ± 0.5 µmol/L; GLU: 7.6 ± 0.7 mmol/L) as well as naïve animals (TBIL: 5.3 ± 0.4 µmol/L; GLU: 10.3 ± 2.2 mmol/L) and ^225^Ac-treated naïve animals (TBIL: 5.4 ± 0.5 µmol/L; GLU: 11.3 ± 1.2 mmol/L).

### IHC and HE Staining

**Fig. 6:**
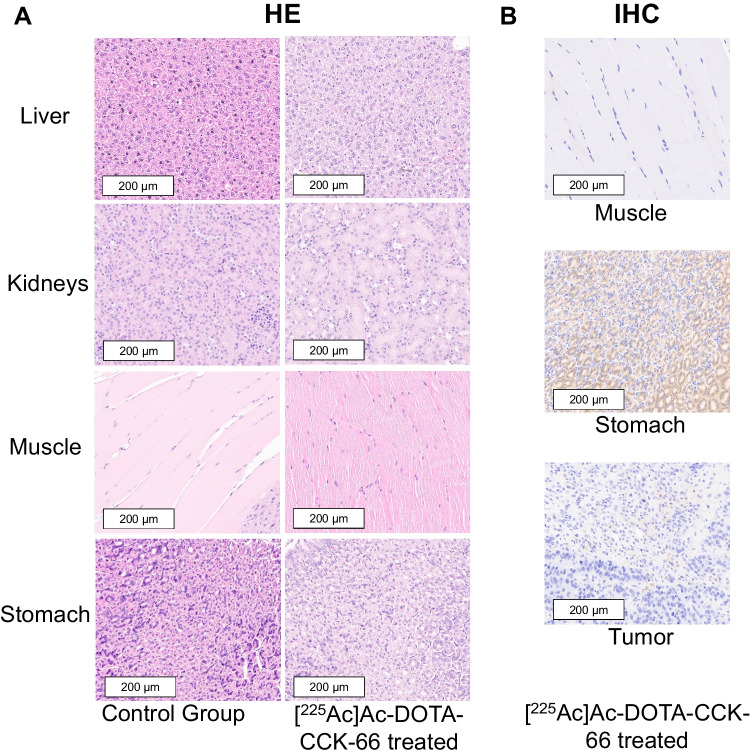
Representative images of (**A**) HE as well as (**B**) IHC stains of different organs isolated from the untreated control (10–17 d after injection) and [^225^Ac]Ac-DOTA-CCK-66-treated animals (49–64 d after injection). All images are shown at a 20-fold magnification. Brown staining indicates CCK-2R positivity

IHC as well as HE staining were performed with different organs collected from animals of the untreated control (stomach, kidney, liver, and muscle; 10–17 d after injection; *n* = 3) as well as [^225^Ac]Ac-DOTA-CCK-66 treated tumor-bearing animals (tumor, stomach, kidney, liver and muscle; 49–64 d after injection; *n* = 3) (Fig. 6, Supplemental Fig. 3).Organs of only three mice per cohort were evaluated. While the stomach displayed high CCK-2R expression in IHC stains for both cohorts evaluated, CCK-2R expression in the ^225^Ac-treated tumor was weak. CCK-2R-negative organs (liver, kidneys and muscle) did not show any accumulation of the CCK-2R directed antibody. The HE stains showed no difference among the control and [^225^Ac]Ac-DOTA-CCK-66 treated animals for all organs evaluated.

## Discussion

Despite progress in the treatment of MTC, current 10-year survival rates demand improved therapeutic approaches. Recent studies suggest that targeting the CCK-2R by radiolabeled compounds could improve treatment outcomes for MTC. Moreover, such compounds could also be used in other cancer types expressing CCK-2R, such as stromal ovarian cancer, astrocytoma and small cell lung cancer, which indicates a promising clinical value for CCK-2R ligands. Particularly the latter could be of high interest for RLT, as small cell lung cancer is very aggressive and conventional treatment options are scarce, thus resulting in a 5-year survival rate of less than 7% [27]. In a previous study, we introduced DOTA-CCK-66, a metabolically more stable CCK-2R-targeted peptide with comparable as well as improved in vivo characteristics to currently clinically tested minigastrin analogs such as DOTA-MGS5 and DOTA-PP-F11N. Initial PET/CT images of patients suffering from MTC confirmed the clinical value of [^68^Ga]Ga-DOTA-CCK-66 and warrants the further development of a therapeutic analog [24, 25]. Therefore, our presented study goal was to elucidate the therapeutic efficacy of DOTA-CCK-66 labeled either with ^225^Ac for TAT or ^177^Lu for RLT.

The log*D*_7.4_ value of [^225^Ac]Ac-DOTA-CCK-66 (log*D*_7.4_ = − 3.04 ± 0.05) presented to be in a similar and slightly lower range than that of its previously published [^67^Ga]Ga- (log*D*_7.4_ = − 3.01 ± 0.07; [24]) and [^177^Lu]Lu-labeled (log*D*_7.4_ = − 2.60 ± 0.07; [24]) analogs, respectively, which indicates favorable hydrophilic properties of the compound. In vitro stability of [^225^Ac]Ac-DOTA-CCK-66 in human serum was high over a time span of 10 d, confirming sufficient complex stability of [^225^Ac]Ac-DOTA. In addition, a similar stability was found for [^225^Ac]Ac-DOTA-CCK-66 and [^177^Lu]Lu-DOTA-CCK-66 when incubated in human serum at 37 °C for 3 d (91.3 ± 0.3% versus 90.8 ± 0.9%, *p* = 0.35) [24]. A limitation of this study is, that the stability data collected only depict the stability of the ^225^Ac-DOTA complex, not that of its daughter nuclides.

[^68^Ga]Ga-DOTA-CCK-66 PET/CT imaging studies of AR42J tumor-bearing animals assigned to the control group cohort underlined previously published data [24], displaying high tumor uptake while activity uptake in off-target tissue remained low.

Both single-dose administration of [^225^Ac]Ac-DOTA-CCK-66 (37 kBq) and [^177^Lu]Lu-DOTA-CCK-66 (37 MBq) revealed a significant impact on tumor growth inhibition, when compared to the control. In addition, the mean survival of animals treated with [^177^Lu]Lu-DOTA-CCK-66 and [^225^Ac]Ac-DOTA-CCK-66, respectively, was observed to be increased by 3- and 4.5-fold, respectively, when compared to the control group animals, indicating a substantial therapeutic efficacy of both compounds. As expected, the higher linear energy transfer in combination with a higher particle energy and lower particle path length of α-emitters (e.g. ^225^Ac), when compared to β^–^-emitters (e.g. ^177^Lu) [28–30], led to a 1.5-fold increase in mean survival rate of animals treated with [^225^Ac]Ac-DOTA-CCK-66 compared to [^177^Lu]Lu-DOTA-CCK-66. Despite noticeable effects observed after [^177^Lu]Lu-DOTA-CCK-66 and [^225^Ac]Ac-DOTA-CCK-66 RLT, the small animal cohorts tested are a limitation of this preliminary study.

Our study indicates the theranostic potential of DOTA-CCK-66 labeled with either ^68^Ga/^177^Lu or ^68^Ga/^225^Ac as theranostic pairs. This finding is a great advantage of our compound DOTA-CCK-66 compared to currently clinical applied diagnostic tools for MTC, such as the gold standard [^18^F]F-DOPA, which have no therapeutic analog available [31, 32]. Radiotheranostics, in our case DOTA-CCK-66, can provide patients with personalized health care options. CCK-2R positive lesions identified by [^68^Ga]Ga-DOTA-CCK-66 PET/CT in MTC patients could make them eligible for RLT or TAT with ^177^Lu or ^225^Ac-labeled DOTA-CCK-66, respectively.

Previously published studies by *Qin* et al. as well as *Grzmil* et al. revealed that treatment with ^177^Lu- (60 MBq) and ^225^Ac-labeled DOTA-PP-F11N (60 kBq), respectively, led to an increase in mean survival of 1.4- and 2-fold, respectively, when compared to the control group cohort [6, 7]. It has to be mentioned that a different animal model (CD-1 nude mice) and cell line (A431/CCKBR^+^) was used in their studies, which is why comparisons between our study and their studies have to be drawn with caution. Nevertheless, previously performed biodistribution studies of [^177^Lu]Lu-DOTA-PP-F11N and [^177^Lu]Lu-DOTA-CCK-66 using the same parameters (AR42J-tumor bearing CB17-SCID mice, same activity and precursor amounts) revealed significantly higher activity levels in the tumor at 24 h p.i. for the latter (1.88 ± 0.82%ID/g versus 8.56 ± 1.08). This supports our hypothesis of an enhanced therapeutic efficacy of DOTA-CCK-66 compared to DOTA-PP-F11N [24, 33].

Higher activity levels for [^177^Lu]Lu-DOTA-CCK-66 (compared to [^177^Lu]Lu-DOTA-PP-F11N) were also observed in the stomach, endogenously expressing the CCK-2R [34], which can lead to adverse effects when using ^177^Lu-based and ^225^Ac-based treatment, respectively. Therefore, the weight of all tumor-bearing animals was monitored regularly, and none of the mice reached a continuous or critical weight loss of more than 20%. Mean weight slowly increased for all animals from the three cohorts evaluated, which did not indicate any signs of malnutrition. However, it has to be mentioned that the increasing weight of the tumor also contributes to the mean body weight of the animals.

Furthermore, blood samples of ^177^Lu- and ^225^Ac-treated naïve mice in contrast to tumor-bearing animals, as well as the non-treated control cohort, were analyzed to elucidate potential side-effects. Both ALB as well as TP blood levels, considered nutritional laboratory markers for malnutrition [35, 36], were found to be within a similar range (TP: 41–55 g/L; ALB: 31–44 g/L) for all cohorts evaluated, which supports a healthy nutrition of all animals from the experiment. In addition, laboratory markers for renal function, namely BUN, K^+^, Na^+^, and Ca^2+^ blood levels [37–40], were found to be in a similar range within the five different animal cohorts examined (BUN: 5.8–7.9 mmol/L; K^+^: 5.4–6.2 mmol/L; Na^+^: 150–157 mmol/L; Ca^2+^: 2.4–3.3 mmol/L), which does not indicate any signs of renal malfunction at this point. It needs to be mentioned, that nephrotoxicity is known to develop late (> 6 months) after treatment, thus a long-term toxicity study is required to fully asses treatment safety [41].

In order to investigate potential liver toxicity, serum markers (ALT, AST, ALP, and TBIL) were analyzed [42]. While mean AST and TBIL concentrations of tumor-bearing ^225^Ac-treated (AST: 1713 ± 264 U/L; TBIL: 11.8 ± 3.9 µmol/L) and control group animals (AST: 516 ± 68 U/L; TBIL: 8.8 ± 2.5 µmol/L) were found to be noticeably elevated, those of ^225^Ac-treated naïve mice (AST: 298 ± 187 U/L; TBIL: 5.4 ± 0.5 µmol/L) and ^177^Lu-treated naïve (AST: 115 ± 77 U/L; TBIL: 5.3 ± 0.4 µmol/L) as well as ^177^Lu-treated tumor-bearing animals (AST: 269 ± 69 U/L; TBIL: 5.4 ± 0.5 µmol/L) presented to be lower. Elevated AST and TBIL levels can be mainly attributed to tumor burden, as tumor-bearing cohorts, in general, displayed similar or higher serum concentrations than naïve animals. However, further experiments and larger animal groups would be required to confirm this assumption. For both ALT (^225^Ac-treated tumor-bearing animals: 345 ± 166 U/L; all other cohorts: 35–87 U/L) and ALP (^225^Ac-treated naïve animals: 76 ± 14 U/L; all other cohorts: 19 to 32 U/L) values, one out of five cohorts presented elevated serum concentrations, whereas all other cohorts displayed mean values within a comparable range. Further experiments are required to elucidate potential explanations for this observation. However, in general, blood toxicity studies did not indicate an impeded liver function upon [^177^Lu]Lu- or [^225^Ac]Ac-DOTA-CCK-66 treatment.

IHC imaging of animals assigned to the control group (*n* = 3) as well as to the [^225^Ac]Ac-DOTA-CCK-66 treated tumor-bearing cohort (*n* = 3) confirmed endogenous CCK-2R expression of the stomach. However, only a weak CCK-2R signal in tumor tissues of ^225^Ac-treated animals was detected. This observation can be attributed to the necrotic tumor volume of the animals at the end-point of the study in combination with potentially reduced receptor expression levels after ^225^Ac-treatment. Flow cytometry analysis of the AR42J cells used for inoculation confirmed CCK-2R expression of the cell line. In addition, PET/CT imaging of tumor-bearing control group animals verified a CCK-2R-specific uptake of [^68^Ga]Ga-DOTA-CCK-66. Not surprisingly, no CCK-2R expression was observed in the liver, the muscle, or the kidneys, as these organs do not overexpress the CCK-2R. In addition, HE stains of all tissues evaluated displayed no structural difference between the control group and the ^225^Ac-treated animals. Thus, no toxcicity delivered by ^225^Ac-directed TAT to healthy organs could be found. The main focus of this study was to evaluate the possibilities and challenges of ^225^Ac-TAT for the treatment of MTC. Thus, no histological analysis of tissues from ^177^Lu-treated animals was performed, which is a limitation of this study.

The promising data of this preliminary study, which reflects the high therapeutic potential of CCK-2R directed ^177^Lu-RLT and ^225^Ac-TAT, should encourage future comparative preclinical evaluation of our novel compounds [^177^Lu]Lu-DOTA-CCK-66 and [^225^Ac]Ac-DOTA-CCK-66 in regard to biodistribution, dosimetry, dose-dependant response and long term-toxicity studies, to further pave the way for first in-human clinical applications in MTC patients.

## Conclusion

We could successfully demonstrate a substantial increase in mean survival of AR42J tumor-bearing mice upon treatment with the minigastrin derivative, DOTA-CCK-66, when either ^177^Lu- or ^225^Ac-labeled. As expected, the latter compound displayed a higher therapeutic efficacy. In addition, no indication of toxicity to the kidneys, liver, or stomach upon ^177^Lu- as well as ^225^Ac-treatment was observed. The minigastrin analog, DOTA-CCK-66, is a promising clinical candidate for PET/CT imaging. Moreover, initial treatment studies should be safe for application in cancer patients, which, however, has to be confirmed in a clinical setting first.

## Supplementary Information

Below is the link to the electronic supplementary material.

Supplementary file1 (DOCX 1.51 MB)

## Data Availability

The datasets generated during and/or analysed during the current study are available from the corresponding author on reasonable request.
