# Supplementary material for: Preclinical evaluation of 225Ac-labeled minigastrin analog DOTA-CCK-66 for Targeted Alpha Therapy
Source: Eur J Nucl Med Mol Imaging. 2024 Oct 11;52(2):458–68. doi: 10.1007/s00259-024-06927-z (PMC11732879; doi:10.1007/s00259-024-06927-z)
Supplement: Supplementary file 1 — Supplementary file1 (DOCX 1.51 MB) [file 259_2024_6927_MOESM1_ESM.docx]

**– Supplemental Materials –**

Nadine Holzleitner^1^, Meryl Vilangattil^2^, Abir Swaidan^2^, Clara Diaz Garcia-Prada^2^, Marco F. Taddio^2^, Pauline Jeanjean^2^, Christine E. Mona^2^, Constantin Lapa^3^, Angela Casini^1,4^, Thomas Günther^1,5^ and Giuseppe Carlucci^2^

^1^ Chair of Pharmaceutical Radiochemistry, Department of Chemistry, School of Natural Sciences, Technical University of Munich, Walther-Meissner-Str. 3, 85748, Garching, Germany

^2^ Department of Molecular and Medical Pharmacology, University of California Los Angeles, Los Angeles, USA

^3^ Nuclear Medicine, Faculty of Medicine, University of Augsburg, Augsburg, Germany

^4^ Chair of Medicinal and Bioinorganic Chemistry, Department of Chemistry, School of Natural Sciences, Technical University of Munich, Garching, Germany

^5^ Molecular Imaging Program at Stanford (MIPS), Department of Radiology, School of Medicine, Stanford University, Stanford, CA, USA

**Co-corresponding authors:**

| Nadine Holzleitner  Phone: +49.89.289.12203  E-Mail: [nadine.holzleitner@tum.de](mailto:nadine.holzleitner@tum.de)  ORCID: <https://orcid.org/0000-0001-8258-3526> | Giuseppe Carlucci  +1.310.794.7638  [GCarlucci@mednet.ucla.edu](mailto:GCarlucci@mednet.ucla.edu)  <https://orcid.org/0000-0001-7494-335X> |
| --- | --- |

**Address of corresponding authors:**

| Technical University of Munich,  Chair of Pharmaceutical Radiochemistry,  Walther-Meissner-Str. 3  85748 Garching  GERMANY | University of California, Los Angeles  Biomedical Cyclotron Facility  780 Westwood Plaza,  90024 Los Angeles, CA USA |
| --- | --- |

**Labeling Procedures**

**^68^Ga-Labeling:**

^68^Ga-labeling was conducted according to a semi-automated synthesis protocol. Thus, ^68^GaCl_3_ was eluted from a GalliaPharm^®^-generator (Eckert&Ziegler, Atlanta, GA, USA) using HCl (5 mL, 0.1 M) and loaded onto a PS-H^+^ cartridge preconditioned with HCl (3 mL, 1 M). Afterward, [^68^Ga]gallium was eluted into the reaction vessel, containing sodium ascorbate buffer (10 µL, 10 mg/mL), (4-(2-hydroxyethyl)-1-piperazineethanesulfonic acid (HEPES) buffer (1.4 mL, 1.5 M) and peptide precursor (10 µg, 7.1 nmol, 10 µL dissolved in dimethyl sulfoxide (DMSO)), using saline (5 mL). The reaction mixture was heated to 90°C for 5 min and loaded onto a C-18 cartridge. After two subsequent washing steps with H_2_O (reaction vessel: 1×3 mL, cartridge: 1×8 mL), [^68^Ga]Ga-DOTA-CCK-66 was eluted from the cartridge using EtOH (500 µL) and 0.9% saline solution (3 mL). Radiochemical purity (RCP) was confirmed via radio reversed phase (RP) thin-layer chromatography (TLC, instant TLC-silica gel (SG) strips, 1 m ammonium acetate/methanol, 1/1, *v*/*v*) using a *mini*Gita radio-TLC scanner (Elysia-raytest GmbH, Straubenhardt, Germany).

**^177^Lu-Labeling:**

Manual ^177^Lu-labeling of the peptide precursor was carried out according to established protocols. Therefore, [^177^Lu]LuCl_3_ (533 MBq, 60 µL, Oak Ridge National Laboratory, Oakridge, TN, USA), DOTA-CCK-66 (36 µg, 26 nmol, 36 µL, dissolved in DMSO, 1 mg/mL) and 2,5-hydroxybenzoic acid buffer (304 µL, 10 mg/mL 2,5-dihydroxybenzoic acid, NaOAc, 0.4 M, pH=4.8, freshly prepared on the day of the experiment) were mixed and heated to 95°C for 15 min. Afterward, RCP was analyzed via RP-TLC (50 mM EDTA) on a Bioscan AR-2000 radio-TLC scanner (Washington, DC, USA).

**^225^Ac-Labeling:**

^225^Ac-labeling was conducted according to a manual synthesis protocol. Thus, a reaction solution of [^225^Ac]AcCl_3_ (296 kBq, 18 µL, Oak Ridge National Laboratory) dissolved in HCl (0.1 M), peptide precursor (DOTA-CCK-66, 2 µg, 1.4 nmol, 2 µL, dissolved in DMSO, 1 mg/mL, activity actinium (A_Ac_)/4≙m(peptide)) and 2,5-hydroxybenzoic acid buffer (10 µL, A_Ac_×1.2≙V(buffer), 10 mg/mL 2,5-dihydroxybenzoic acid, NaOAc, 1 M, freshly prepared on the day of the experiment) was prepared and filled with H_2_O to a total reaction volume of 200 µL. Subsequently, the reaction solution was heated to 90°C for 30 min. RCP was determined using radio-RP-TLC (50 mM EDTA, AR-2000 radio-TLC scanner, Bioscan, WinScan software). TLC plates were analyzed 24 h after processing to wait for secular equilibrium of ^225^Ac with its γ-emitting daughter nuclides (^221^Fr and ^213^Bi).

**In Vitro Experiments**

**Cell Culture**

CCK-2R expressing rat pancreatic cancer cells AR42J (ATCC, Manassas, VA, USA) were cultivated in monolayers in Petri dishes at 37°C in a humidified atmosphere (5% CO_2_, 20% O_2_). As a nutrient medium, F-12K medium was used, supplemented with 10% FBS (Omega Scientific, Giza, Egypt) and 6 mL penicillin-streptomycin solution (10.000 U/mL). A Trypsin-EDTA (0.25%) solution was applied to detach the cells for passaging. Cell counting was conducted with a Vi-CELL^TM^ XR cell counter (Beckman Coulter GmbH, Brea, CA, USA). In addition, all operations under sterile conditions were accomplished using a LabGard^®^ biological safety cabinet (Class II, Type A2, NuAire, Plymouth, MA, USA)*.*

**Lipophilicity Studies**

Lipophilicity (depicted as *n*-octanol/phosphate-buffered saline (PBS, pH=7.4) solution distribution coefficient, log*D*_7.4_) was determined. Thus, [^225^Ac]Ac-DOTA-CCK-66 (~56 kBq, 10 μL, in HCl, 0.01 N) were dissolved in a mixture (1 mL, 1/1, *v/v*) of *n-*octanol and PBS. The suspension was vortexed in a reaction vial for 3 min at room temperature. Afterward, the vial was centrifuged for 5 min at 7830 rpm (Centrifuge 5430, Eppendorf SE, Enfield, CT, USA). 200 μL aliquots of both layers were separated and, after 24 h, measured by a WIZARD^2®^ 2480 Automatic γ-Counter (Perkin Elmer Inc., Waltham, MA, USA). The experiment was repeated seven times.

**In Vitro Stability Studies in Human Serum**

To determine the in vitro stability of [^225^Ac]Ac-DOTA-CCK-66, the ^225^Ac-labeled peptide (~259 kBq, 4 nmol, 50 μL, in HCl, 0.01 N) was added to human serum (200 μL, healthy donor) and incubated at 37°C for several days (1-10 d). At each time point, small samples (~5 μL) were collected and analyzed via radio-RP-TLC. The experiment was repeated three times.

**Flow Cytometry**

The first 5.0×10^5^ AR42J cells were prepared per sample. Thereafter, cells were washed with 400 µL of a 0.5% bovine serum albumin (BSA) in Dulbecco's Phosphate-Buffered Saline (DPBS) solution and centrifuged for 5 min (450 g) at 4°C. Meanwhile, a 1 µg/mL solution of polyclonal CCK antibody (1 mg/mL in PBS, pH=7.5, supplemented with 50% glycerol, PA5-103116, rabbit, ThermoFisher Scientific Inc., Waltham, MA, USA) diluted in 0.5% BSA in DPBS was prepared. Afterward, 50 µL of the diluted antibody (stained condition) or PBS (unstained condition) were added to the cells, briefly vortexed, and incubated in the dark at 4°C for 20-30 min. Subsequently, cells were washed with 400 µL of 0.5% BSA in DPBS, and centrifugation was repeated as described above. The supernatant was decanted from the cells, and the washing step was repeated twice. Meanwhile, the secondary APC anti-mouse IgG1 antibody (Clone RMG1-1, 0.2 mg/µL, BioLegend, San Diego, CA, USA) was diluted to a final concentration of 2.5µg/mL using 0.5% BSA in DPBS solution. Afterward, 50 µL of diluted secondary antibody (stained condition) and PBS (unstained condition) were added to the samples. A brief vortex step was followed by incubating the samples at 4°C in the dark for 20-30 min. After that, three subsequent washing steps (400 µL 0.5% BSA in DPBS) were carried out, followed by centrifugation (5 min, 450 g, 4°C) and decantation. The cell pellet was dispensed in 400 µL PBS and measured on a 5-laser LSR II cytometer (BD Biosciences, Heidelberg, Germany) and analyzed using FlowJo^TM^ (ThreeStar, BD Biosciences) software.

**In vivo Experiments**

**Toxicity Studies**

Blood samples were collected retro-orbitally using a glass capillary tube once mice reached one of the pre-defined endpoints. Afterward, the blood samples were centrifuged at 6.000 rpm for 10 min (Centrifuge 5417R, Eppendorf SE, Enfield, CT, USA) and the supernatant was filled into a preventive care profile plus rotor (GLU, ALB, ALP, ALT, AST, BUN, Ca^2+^, Cl^-^, CRE, GLOB, K^+^, Na^+^, TBIL, *^t^*CO_2_, TP). Sample analysis was conducted according to standard operating procedures of VetScan VS2 (Abaxis, Union City, CA, USA).

**IHC Staining**

Tissues were fixed in 10% formalin for 48 h and stored in 70% ethanol until radioactivity decayed (>10 half-lives of the respective radionuclide). Then, paraffin-embedded samples were sectioned in the center of each probe to obtain tissue slices. Afterward, automated detection was performed stepwise based on protocol F using a Bond Polymer Refine Detection kit (Leica Biosystems, DS9800, Deer Park, IL, USA) on a Leica Bond RX processor. First, the Leica Bond RX routine factory-based “Bake and Dewax” protocol was carried out to remove residual paraffin from the slides. Heat-induced antigen retrieval was performed at 100 °C for 20 min using an ER2 (BOND Epitope Retrieval Solution 2, Leica Biosystems, AR9640) buffer. Afterward, endogenous peroxidase activity was blocked (Refine Detection Kit Peroxide Block, 5 min), followed by three consecutive washing steps (Bond Wash buffer). Thereafter, slices were incubated for 60 min with a polyclonal rabbit IgG CCK antibody (PA1-37267, dilution: 1/200, ThermoFisher Scientific Inc., Waltham, MA, USA). After three consecutive washing steps (Bond Wash buffer), secondary staining was performed using the Dakocytomation Envision System labeled polymer horseradish peroxidase anti-rabbit (Agilent K4003, ready to use) for 10 min. Afterward, washing was repeated (3×2 min, Bond wash buffer), and the slices were incubated with polymer for another 8 min. After incubation, another five consecutive washing steps using a Bond wash buffer and a washing step applying deionized water were completed. After that, slices were visualized via incubation with a Mixed Diaminobenzidine (DAB) Refine detection kit applied twice (1×5 s; 1×10 min) according to the manufacturer’s instructions. Subsequently, sections were washed with deionized water (3×), counterstained with hematoxylin for 10 min, and washed with Bond Wash buffer (3×) as well as deionized water (1×). In the last step, slides were dehydrated in a series of alcohols (95% ethanol, 2×10 s; 100% ethanol, 2×10 s), cleared with his clear (2×10s), and mounted with Permount. To acquire IHC images, all slides were scanned digitally at 20× magnification using ScanScope AT (Leica Biosystems, Vista).

**Supplemental Data**

**Supplemental Table 1.** Amount of intact peptide of [^225^Ac]Ac-DOTA-CCK-66, determined in human serum via incubation at 37°C for 1 to 10 d, as analyzed by analytical radio-RP-TLC (50 mM EDTA). Data are expressed as mean±SD (*n*=3).

| **Time (d)** | **Amount of intact peptide (%)** |
| --- | --- |
| Quality Control | >99% |
| 1 | 93.0±0.5 |
| 2 | 91.8±1.0 |
| 3 | 91.3±0.3 |
| 7 | 82.2±3.4 |
| 10 | 83.4±10.3 |

**Supplemental Table 2.** Tumor volume (mm^3^) over time (0-49 d) of AR42J-tumor bearing 394-NOD SCID mice, injected either with [^68^Ga]Ga-DOTA-CCK-66 (control cohort, 1.1 MBq, 29 pmol), [^225^Ac]Ac-DOTA-CCK-66 (37 kBq, 178 pmol), or [^177^Lu]Lu-DOTA-CCK-66 (37 MBq, 1.7 nmol) on day 0 of the experiment. Data are expressed as mean±SD (*n*=5).

|  | **Tumor volume (mm^3^)** | | |
| --- | --- | --- | --- |
| Time (d) | Control cohort  ([^68^Ga]Ga-DOTA-CCK-66) | [^225^Ac]Ac-DOTA-CCK-66 treatment cohort | [^177^Lu]Lu-DOTA-CCK-66 treatment cohort |
| 0 | 200±100 | 150±80 | 170±70 |
| 3 | 460±230 | 170±110 | 110±40 |
| 7 | 890±440 | 130±60 | 110±50 |
| 10 | 1,410±570 | 100±60 | 120±60 |
| 14 | - | 100±50 | 210±60 |
| 17 | - | 100±50 | 250±80 |
| 21 | - | 90±60 | 390±250 |
| 24 | - | 130±70 | 290±110 |
| 28 | - | 130±80 | 440±380 |
| 31 | - | 160±90 | 550±420 |
| 35 | - | 240±130 | - |
| 38 | - | 470±280 | - |
| 42 | **-** | 740±430 | - |
| 45 | - | 1,040±690 | - |
| 49 | - | 1,600±830 | - |

**Supplemental Table 3.** Body weight over time (0-49 d) of AR42J-tumor bearing 394-NOD SCID mice, injected either with [^68^Ga]Ga-DOTA-CCK-66 (control cohort, 1.1 MBq, 29 pmol), [^225^Ac]Ac-DOTA-CCK-66 (37 kBq, 178 pmol), or [^177^Lu]Lu-DOTA-CCK-66 (37 MBq, 1.7 nmol) on day 0 of the experiment. Data are expressed as mean±SD (*n*=5).

|  | **Body weight (g)** | | |
| --- | --- | --- | --- |
| Time (d) | Control cohort  ([^68^Ga]Ga-DOTA-CCK-66) | [^225^Ac]Ac-DOTA-CCK-66 treatment cohort | [^177^Lu]Lu-DOTA-CCK-66 treatment cohort |
| 0 | 21.8±2.3 | 20.4±1.5 | 25.8±0.7 |
| 3 | 20.6±2.1 | 20.6±1.4 | 25.8±1.0 |
| 7 | 22.2±2.1 | 20.8±1.5 | 26.4±0.5 |
| 10 | 22.0±2.6 | 21.2±1.6 | 25.8±1.2 |
| 14 | 20.5±0.5 | 20.8±1.2 | 26.0±1.1 |
| 17 | - | 21.2±1.2 | 26.0±0.9 |
| 21 | - | 21.2±1.2 | 26.6±0.8 |
| 24 | - | 21.4±1.4 | 25.8±0.7 |
| 28 | - | 21.4±1.0 | 25.8±1.0 |
| 31 | - | 21.8±1.2 | 26.8±1.6 |
| 35 | - | 22.2±1.0 | - |
| 38 | - | 23.0±0.6 | - |
| 42 | **-** | 23.0±0.9 | - |
| 45 | - | 23.4±0.8 | - |
| 49 | - | 23.0±1.7 | - |

**Supplemental Table 4.** Blood values of [^225^Ac]Ac-DOTA-CCK-66 treated AR42J tumor-bearing mice compared to [^225^Ac]Ac-DOTA-CCK-66 treated naïve mice and AR42J tumor-bearing control group mice as analyzed by VetScan VS2. Blood samples were collected at the end-point of the experiment (AR42J tumor-bearing mice) or in analogy to [^225^Ac]Ac-DOTA-CCK-66 treated AR42J tumor-bearing mice (naïve mice). Data are expressed as mean±SD (*n*=5).

|  | **Control cohort (AR42J tumor-**  **Bearing mice)** | **[^225^Ac]Ac-DOTA-CCK-66** **treatment cohort (AR42J**  **tumor-bearing mice)** | **[^225^Ac]Ac-DOTA-CCK-66** **treatment cohort (naïve mice)** |
| --- | --- | --- | --- |
| **BUN (mmol/L)** | 5.82±0.45 | 6.08±1.61 | 7.06±0.45 |
| **ALT (U/L)** | 79.2±13.3 | 350±166 | 87.2±53.0 |
| **ALP (U/L)** | 29.6±13.3 | 20.8±8.6 | 75.6±13.5 |
| **AST (U/L)** | 516±68 | 1713±264 | 298±187 |
| **TBIL (µmol/L)** | 8.80±2.48 | 11.8±3.9 | 5.40±0.49 |
| **GLU (mmol/L)** | 5.94±1.94 | 4.46±2.23 | 11.3±1.2 |
| **Ca^2+^ (mmol/L)** | 3.32±0.19 | 2.38±0.04 | 2.44±0.06 |
| **TP (g/L)** | 51.2±3.3 | 45.2±2.3 | 48.0±1.1 |
| **ALB (g/L)** | 40.0±3.0 | 34.0±2.3 | 38.2±1.6 |
| **GLOB (g/L)** | 11.4±0.5 | 11.0±0.9 | 9.80±1.2 |
| **Na^+^ (mmol/L)** | 151±4 | 155±1 | 150±1 |
| **K^+^ (mmol/L)** | 5.94±0.83 | 5.88±0.23 | 5.40±0.39 |
| ***^t^*CO_2_ (mmol/L)** | 22.0±2.8 | 21.6±2.9 | 19.0±1.4 |

**Supplemental Table 5.** Blood values of [^177^Lu]Lu-DOTA-CCK-66 treated AR42J tumor-bearing mice (*n=*5) compared to [^177^Lu]Lu-DOTA-CCK-66 (*n=*4) treated naïve mice as analyzed by VetScan VS2. Blood samples were collected at the end-point of the experiment (AR42J tumor-bearing mice) or in analogy to [^177^Lu]Lu-DOTA-CCK-66 treated AR42J tumor-bearing mice (naïve mice).

|  | **[^177^Lu]Lu-DOTA-CCK-66** **treatment**  **cohort (AR42J tumor-bearing mice)** | **[^177^Lu]Lu-DOTA-CCK-66** **treatment**  **cohort (naïve mice)** |
| --- | --- | --- |
| **BUN (mmol/L)** | 7.94±0.80 | 7.43±0.54 |
| **ALT (U/L)** | 60.8±25.4 | 35.3±12.4 |
| **ALP (U/L)** | 19.0±5.3 | 32.0±8.7 |
| **AST (U/L)** | 269±69 | 115±77 |
| **TBIL (µmol/L)** | 5.40±0.49 | 5.25±0.43 |
| **GLU (mmol/L)** | 7.64±0.73 | 10.3±2.2 |
| **Ca^2+^ (mmol/L)** | 2.61±0.20 | 2.69±0.13 |
| **TP (g/L)** | 49.0±0.9 | 52.3±1.1 |
| **ALB (g/L)** | 37.0±1.4 | 40.0±0.0 |
| **GLOB (g/L)** | 12.4±2.1 | 12.3±1.1 |
| **Na^+^ (mmol/L)** | 154±3 | 157±3 |
| **K^+^ (mmol/L)** | 5.98±0.82 | 6.18±0.41 |
| ***^t^*CO_2_ (mmol/L)** | 18.6±3.2 | 21.3±3.0 |

**Analytical Data of DOTA-CCK-66**

**
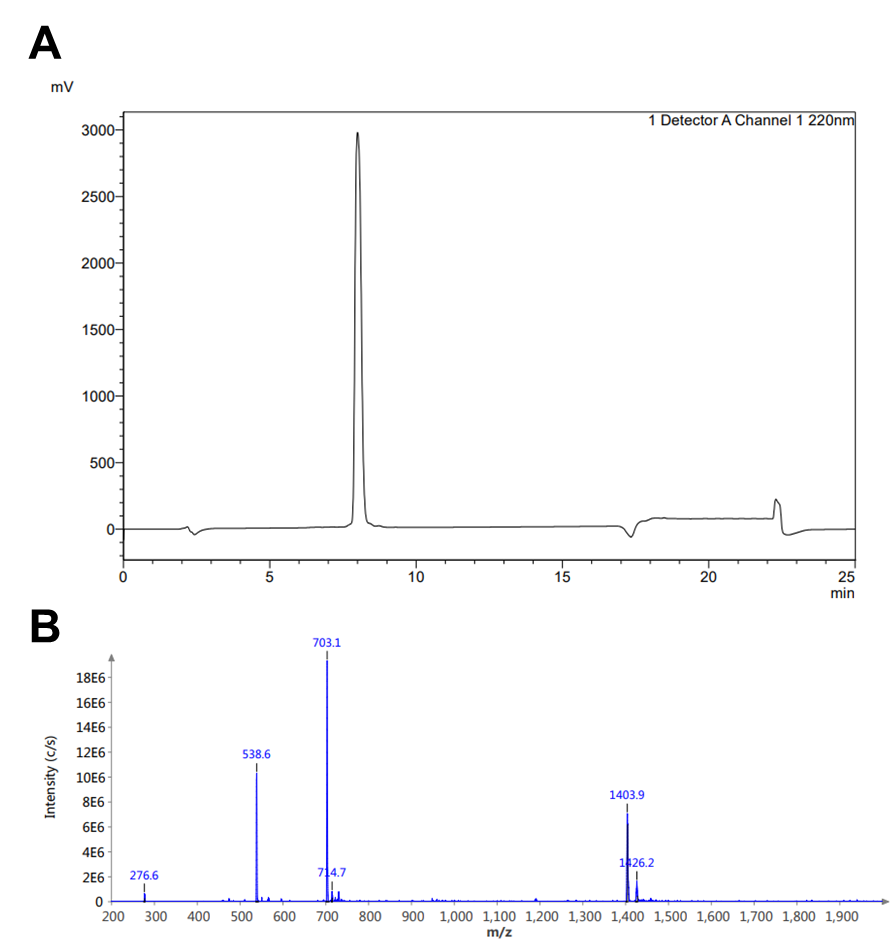
**

**Supplemental Figure 1.** (A) Confirmation of peptide identity and integrity for DOTA-CCK-66 as analyzed by analytical RP-HPLC (MultoKrom 100-5 C18, 5 μm, 125×4.6 mm, CS Chromatographie GmbH, Langerwehe, Germany; 10→90% MeCN in H_2_O + 0.1% TFA in 15 min). (B) Mass spectrum of DOTA-CCK-66.

***DOTA-CCK-66.*** RP-HPLC (10→90% MeCN in H_2_O with 0.1% TFA, 15 min, λ = 220 nm): *t*_R_ = 8.0 min, *K*’ = 3.00; MS (ESI, positive): m/z calculated for C_67_H_96_N_12_O_21_: 1405.6, found: m/z = 1403.9 [M+H]^+^, 703.1 [M+2H]^2+^.

**PET/CT Imaging**

**
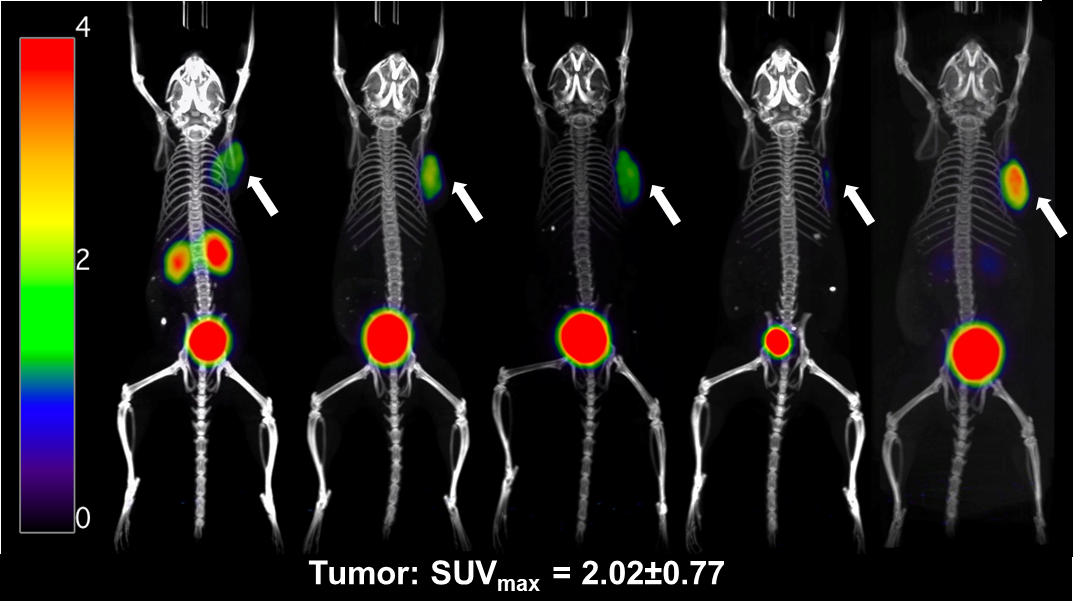
**

**Supplemental Figure 2.** PET/CT scans ([^68^Ga]Ga-DOTA-CCK-66; 1.1 MBq, 30 pmol) of control group AR42J tumor-bearing mice (*n=*5) on day 0 of the experiment. Tumor is indicated with a white arrow.

**Staining Data**

**
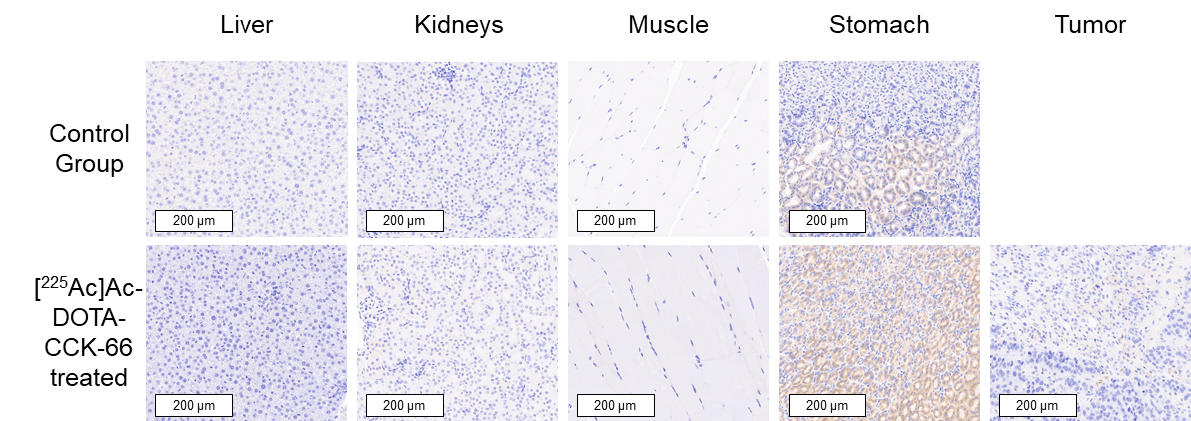
**

**Supplemental Figure 3.** Representative images of IHC stains of different organs isolated from the control group (10-17 d after injection) and [225Ac]Ac-DOTA-CCK-66-treated animals (49-64 d after injection). All images are shown at 20-fold magnification. Brown staining indicates CCK-2R positivity.
